# Laser Therapy of Recurrent Aphthous Ulcer in Patient with HIV Infection

**DOI:** 10.1155/2012/695642

**Published:** 2012-12-30

**Authors:** Bruno Vieira Caputo, Gilberto Araujo Noro Filho, Camila Correia dos Santos, Yugo Okida, Elcio Magdalena Giovani

**Affiliations:** Paulista University (Unip), 1212 Dr. Bacelar Street, Vila Clementino, 04026-002 São Paulo, SP, Brazil

## Abstract

The recurrent aphthous ulcer (RAU) is a pathological change found in the oral mucosa, characterized by painful single or multiple ulcers. The etiologic aspect of RAU is not well understood; however it is known that due to lower CD4 cell counts patients had higher prevalence of these oral lesions, and immunosuppressed patients with HIV are predisposed. Patient FC is African descent, 26 years old, male, HIV + CD4 67 cells/mm^3^, with minor RAU in the upper and lower right side lip, measuring about 4 mm, and major RAU in tongue and the tonsillar pillar measuring 2 cm. The patient was treated with laser therapy with the objective to help reverse the damage and decrease the symptoms. After one week there was remission of the lesions. The laser showed to be an important alternative therapy that promoted analgesic, healing effects and improving the quality of life of patients.

## 1. Introduction

Recurrent aphthous ulcer (RAU) is a common pathological changes found in the oral mucosa, characterized by painful single or multiple ulcers, of poorly understood but much discussed [1]. This is a result of oral epithelium lesion, which typically exposed nerve endings, resulting in pain or soreness especially when eating spicy foods or citrus fruits. The ulcers and erosions may also be local manifestations of systemic diseases. These range from epithelial damage resulting from trauma; an immunological attack as in lichen planus, pemphigoid or pemphigus; damage because of an immune defect as in HIV disease and leukemia; infections such as herpes viruses, tuberculosis, and syphilis; cancer and nutritional defects such as vitamin deficiencies and some gastrointestinal disorders [2]. Some predisposing factors may be identifiable for RAU: stress, trauma, deficiencies of iron, folic acid or vitamin B12, sodium lauryl sulfate (SLS), cessation of smoking, gastrointestinal disorders, and foods allergies. HIV is also considered as a predisposing factor for RAU due to immune deficiency [2, 3]. There is no known laboratory procedure available to establish a definite diagnosis, and histopathological examination of biopsies does not provide a definitive diagnosis. The identification of RAU in clinical practice usually relies on the combination of history and clinical features. Biopsy is only indicated in cases of suspecting another differential diagnosis [4]. Minor aphthous ulcers (Mikulicz Ulcer) are small round or ovoid ulcers 2–4 mm in diameter. They are surrounded by an erythematous halo and are found mainly on the nonkeratinized mucosa of the lips, floor of the mouth or ventral surface of the tongue. They heal in 7 to 10 days and recur at intervals of one to four months leaving little or no evidence of scarring. The major aphthous ulcers (Sutton's Ulcers) are larger, usually about 1 cm in diameter or even larger; of longer duration, heal slowly over 30 to 40 days; of more frequent recurrence and often more painful than minor ulcers. They are found on any area of the oral mucosa, including the keratinized dorsum of the tongue or palate [1, 2].

There is no curative treatment for RAU. The best that can be achieved is to avoid local traumatic precipitants, lessen the pain and duration of ulceration by suppressing the local immune response, and prevent secondary infection [4].

## 2. Case Report

**Figure 1:**
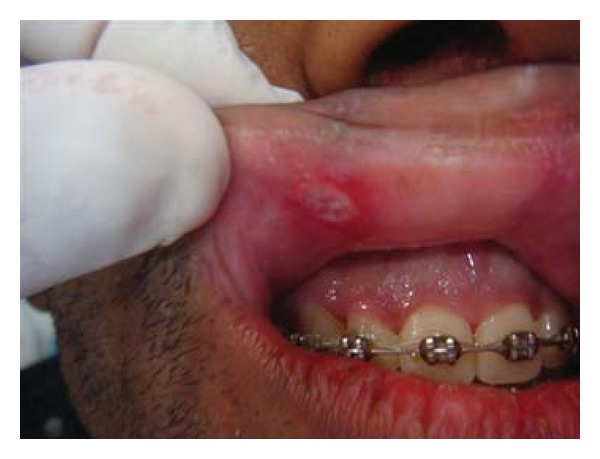
Minor RAU in the upper right side lip.

**Figure 2:**
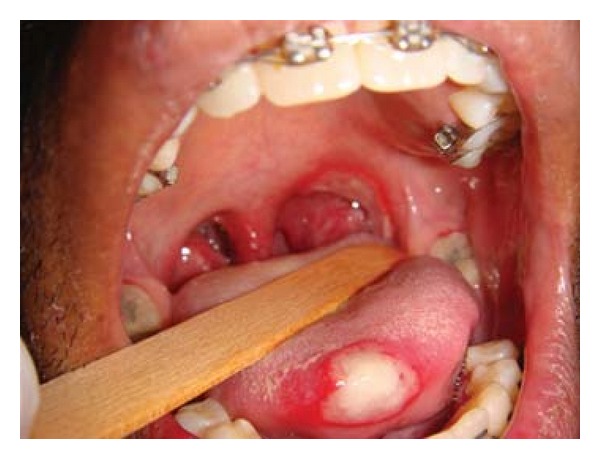
Major RAU in tongue and the tonsillar pillar.

**Figure 3:**
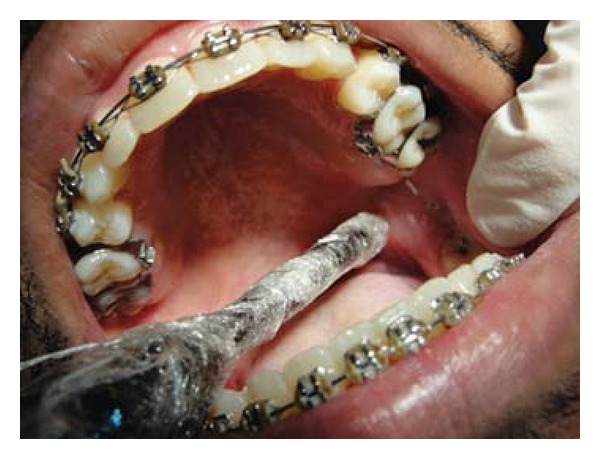
Application of low-intensity laser light GaAlAs in the tonsillar pillar major ulcer.

**Figure 4:**
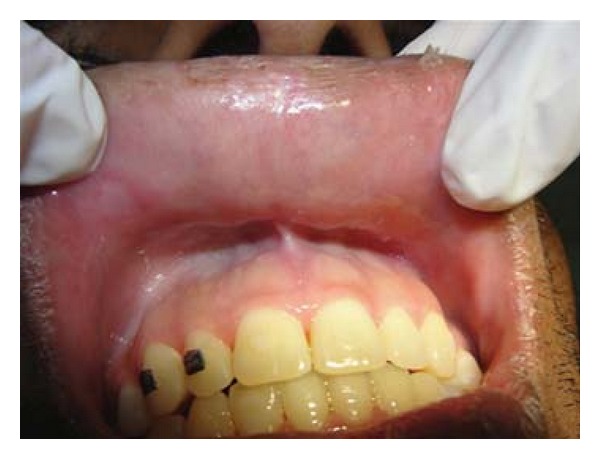
Remission of the lesions after one week of laser application in upper right side lip.

**Figure 5:**
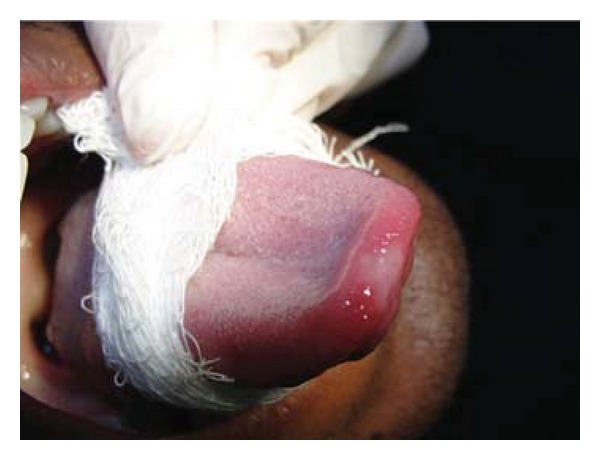
Remission of the lesions after one week of laser application in tongue.

**Figure 6:**
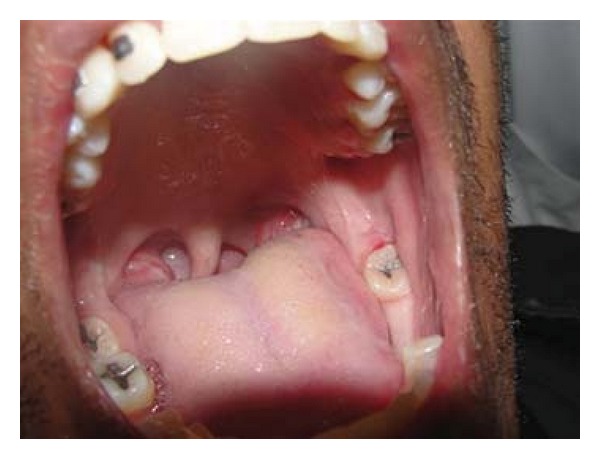
Remission of the lesions after one week of laser application in tonsillar pillar.

Patient FC, African descent, 26 years old, male, HIV+, exposure category man who have sex with men (MSM), last CD4 cell counts 67 cells/mm^3^, virological status 5000 copies/mm^3^, came to the Center for Study and Care of Special Patients(CEAPE), School of Dentistry, Paulista University(UNIP), SP, Brazil, with minor RAU in the upper (Figure 1) and lower right side lip, measuring about 5 mm, and major RAU in tongue and the tonsillar pillar (Figure 2) measuring 2 cm. Reported pain at the site of ulcers and difficulty in eating (swallowing). The patient is HIV positive since 2008 and makes use of antiretroviral therapy (HAART): tenofovir, Kaletra, and 3TC. He reported symptoms of night sweats, diarrhea, fever, arthralgia, myalgia, and weight loss. Due to the medication already used by the patient and the condition of lesions, was advocated as the treatment of RAU the use of a diodo laser Gallium Aluminum Arsenide (GaAlAs) with a wavelength of 660 nm and with a spot size of 0.07 cm² (Figure 3), the laser therapy was released with a power of 0.03 W, a fluence rate of 0.428 W/cm², in continuous mode and irradiation of 4 Joules each point for 2 minutes and 13 seconds and energy Fuency of 57.14 J/cm², being two points per lesion according to their size [5]. The objective was to help reverse the damage and decrease the symptoms. After one week there was remission of the lesions and the patient reported decrease in the pain (Figures 4, 5, and 6). In the 12-month followup the patient was reported as not having the ulcer that he presented in this case.

The case report was approved by the Ethics Committee of Paulista University (643/09), and the patient has signed the informed consent.

## 3. Discussion

The oral alterations in HIV patients are several including more than 40 manifestations, which many times appear as the first manifestations of the disease or, even today, as an important identifier of treatment failure [6]. African descent individuals are distinguished from other groups of patients, because due to social or cultural differences may cause delay in access to services and antiretroviral therapy, they seem to have a HAART failure or a lack of patients collaboration. With the failure of antiretroviral therapy, with CD4 T cells count may decrease, and CD4 less than 200 cells/mm^3^ and high viral antigen burden 3000 copies/mm^3^ increasing the frequency of oral manifestations such as RAU [7]. HIV-associated RAU lesions tend to be more severe and longer lasting and may cause debilitating pain with associated alteration of important oral functions such as speaking, chewing, and swallowing, which ultimately lead to malnutrition, and weight loss, compromise the ability to take medication and seriously interfere with the quality of life [8]. Mouthwash can effectively provide symptomatic relief of ulcers. Steroids are used as rinses only if the patient is unable to apply topical agents directly to ulcers or if lesions cover a larger area. An aqueous preparation of 0.1% or 0.2% triamcinolone, 0.3% hydrocortisone mouth rinse, and dexamethasone elixir 0.5/5.0 mL were all effective when used three or four times per day [8]. The patient required an additional therapy to solve the problem of ulcers. The low-intensity laser therapy cause special effects such as biostimulation, analgesia, and anti-inflammatory. From the laser directly application into the tissue, the healing process such as photobiostimulation favoring an acceleration of the repair process [9–11]. A recent study evaluates the effect of low-level laser on the control of pain and the repair of recurring aphthous stomatitis (RAS). The results revealed that 75% of the patients reported a reduction in pain in the same session after laser treatment, and total regression of the lesion occurred after 4 days; in addition the total regression in the corticoid group was from 5 to 7 days. As a result the use of LLLT demonstrated analgesic and healing effects with regard to RAS [5]. LLLT has a function to improve oral health condition of the patient, allowing the feeding, grooming and giving the time required for the body of the patient to adjust the HAART medication. After one week there was remission of the lesions, with the major aphthous ulcers (Sutton's Ulcers) healing slowly over 30 to 40 days.

## 4. Conclusion 

The use of low intensity laser in RAU provides pain relief, in this case report laser showed to be an important alternative therapy for treatment of minor and major ulcers in HIV patient, improving symptoms, assisting in the regression of lesions, and improving the quality of life of this patient.

## References

[1] Natah SS, Konttinen YT, Enattah NS, Ashammakhi N, Sharkey KA, Häyrinen-Immonen R (2004). Recurrent aphthous ulcers today: a review of the growing knowledge. *International Journal of Oral and Maxillofacial Surgery*.

[2] Scully C, Felix DH (2005). Oral medicine—update for the dental practitioner. Aphthous and other common ulcers. *British Dental Journal*.

[3] MacPhail LA, Greenspan JS (1997). Oral ulceration in HIV infection: investigation and pathogenesis. *Oral Diseases*.

[4] Scully C, Porter S (2008). Oral mucosal disease: recurrent aphthous stomatitis. *British Journal of Oral and Maxillofacial Surgery*.

[5] de Souza TOF, Martins MAT, Bussadori SK (2010). Clinical evaluation of low-level laser treatment for recurring aphthous stomatitis. *Photomedicine and Laser Surgery*.

[6] Coogan MM, Greenspan J, Challacombe SJ (2005). Oral lesions in infection with human immunodeficiency virus. *Bulletin of the World Health Organization*.

[7] Alegre M, Dalmau J, Domingo P, Roé E, Alomar A (2007). Successful treatment of major oral aphthous ulcers in HIV-1 infection after highly active antiretroviral therapy. *International Journal of Infectious Diseases*.

[8] Ship JA, Chavez EM, Doerr PA, Henson BS, Sarmadi M (2000). Recurrent aphthous stomatitis. *Quintessence International*.

[9] Giovani EM, Martins RB, Melo JAJ, Tortamano N (2007). Use of GaAlAs laser in the treatment of necrotizing ulcerative periodontitis in patients seropositive for HIV/AIDS. *Journal of Oral Laser Applications*.

[10] Pejcic A, Kojovic D, Kesic L, Obradovic R (2010). The effects of low level laser irradiation on gingival inflammation. *Photomedicine and Laser Surgery*.

[11] Sakurai Y, Yamaguchi M, Abiko Y (2000). Inhibitory effect of low-level laser irradiation on LPS-stimulated prostaglandin E2 production and cyclooxygenase-2 in human gingival fibroblasts. *European Journal of Oral Sciences*.

